# Enhancement of alkaline protease production in recombinant *Bacillus licheniformis* by response surface methodology

**DOI:** 10.1186/s40643-023-00641-8

**Published:** 2023-04-20

**Authors:** Ying Zhang, Jingmin Hu, Qing Zhang, Dongbo Cai, Shouwen Chen, Yonghong Wang

**Affiliations:** 1 State Key Laboratory of Bioreactor Engineering, East China University of Science and Technology, 130 Meilong Road, P.O. Box 329, Shanghai, 20037 China; 2 State Key Laboratory of Biocatalysis and Enzyme Engineering Environmental, Microbial Technology Center of Hubei Province College of Life Sciences, Hubei University, Wuhan, China

**Keywords:** Alkaline protease, Recombinant *Bacillus licheniformis*, Medium optimization, Response surface methodology

## Abstract

Alkaline protease is widely used in the food, detergent, and pharmaceutical industries because of its comparatively great hydrolysis ability and alkali tolerance. To improve the ability of the recombinant *Bacillus licheniformis* to produce alkaline protease, single-factor experiments and response surface methodology (RSM) were utilized to determine and develop optimal culture conditions. The results showed that three factors (corn starch content, soybean meal content, and initial medium pH) had significant effects on alkaline protease production (*P* < 0.05), as determined through the Plackett‒Burman design. The maximum enzyme activity was observed with an optimal medium composition by central composite design (CCD): corn starch, 92.3 g/L; soybean meal, 35.8 g/L; and initial medium pH, 9.58. Under these optimum conditions, the alkaline protease activity of strain BL10::aprE was 15,435.1 U/mL, 82% higher than that in the initial fermentation medium. To further investigate the application of the optimum fermentation medium, the overexpressed strain BL10::aprE/pHYaprE was cultured using the optimized medium to achieve an enzyme activity of 39,233.6 U/mL. The present study achieved the highest enzyme activity of alkaline protease by *B. licheniformis* at the shake-flask fermentation level, which has important application value for large-scale production.

**Graphical Abstract:**

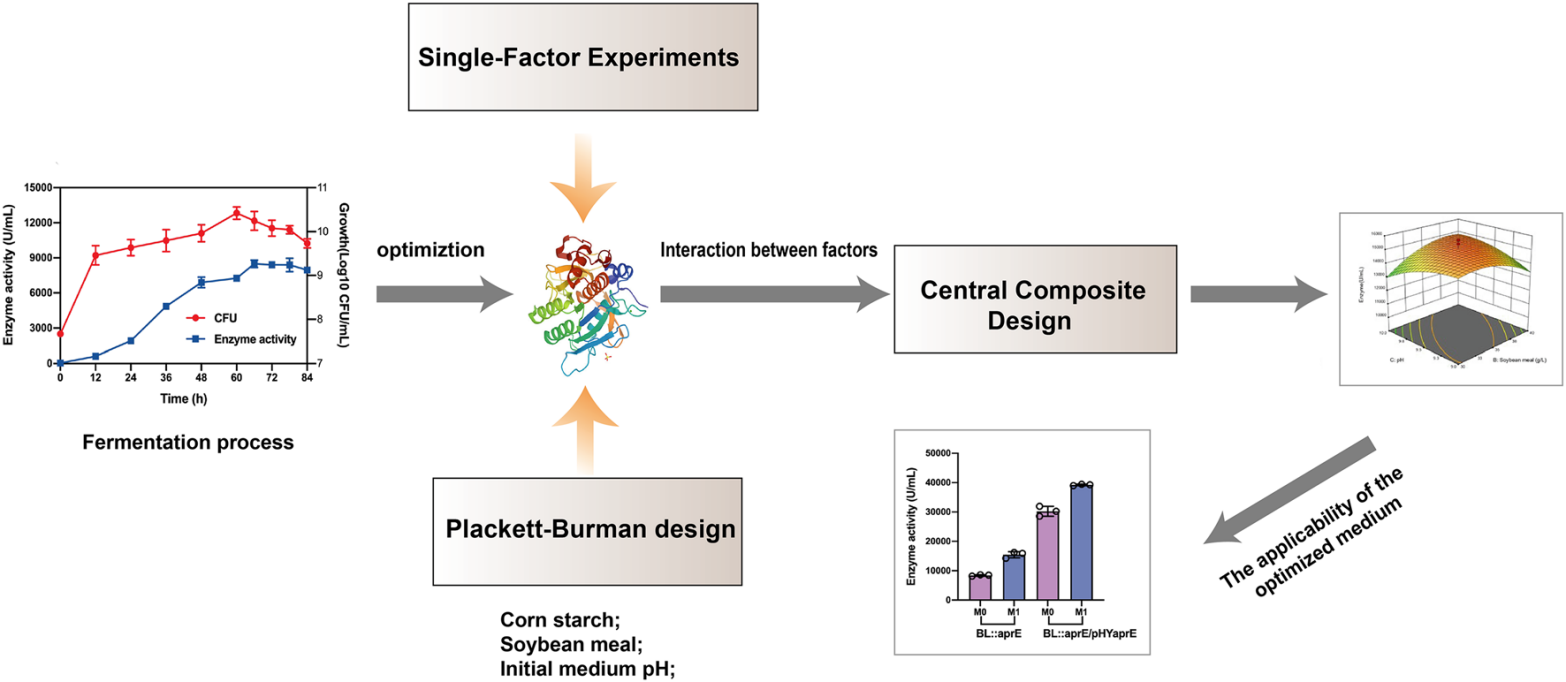

**Supplementary Information:**

The online version contains supplementary material available at 10.1186/s40643-023-00641-8.

## Introduction

Alkaline protease (EC.3.4.21-24), also known as a serine protease, is an endo-peptide protease and is highly active in a neutral-to-alkaline pH range (Sharma et al. 2017). Since alkaline protease possesses excellent features of high catalytic power, specificity, water solubility, and environmental friendliness, it has been extensively used in the fields of food, dairy, silk, detergent, and waste management (Sharma et al. 2019). Notably, protease occupies 60–65% of the global industrial enzyme market (Muhammad et al. 2019) and have shown great commercial application prospects and numerous research activities.

Alkaline proteases that obtained from microorganisms are commonly used for commercial applications due to their high rate of production, simple downstream processing and easy industrial production. In general, *Bacillus* species are efficient alkaline protease producers, such as the commonly used *B. subtilis*, *B. pumilus*, and *B. licheniformis* (Zhou et al. 2020). Among these, *B. licheniformis* has been widely used as a cell factory for protease production in industries due to its ability to secrete abundant protein and biological safety (GRAS) (Li et al. 2019). Alkaline proteases from various microbial sources have relatively varied structures and functions (Shirai et al. 1997). The alkaline protease from *B. clausii* is a highly alkaline enzyme with an optimal pH of 12, and it has tolerance and stability in anionic surfactants and oxidants such as SDS and peroxides, which can be used as additives in detergents to effectively eliminate protein-containing stains in laundry (Joo et al. 2003). However, the production of alkaline proteases derived from *B. clausii* in *B. licheniformis* has received little attention. Recently, studies on alkaline proteases of *B. licheniformis* have mainly focused on the modification of wild bacteria (Zhou et al. 2020, 2019) and the use of molecular biology techniques to improve the yield of proteases (Zhou et al. 2021). Although some achievements have been made, current production still hardly meets the increasing demands of the market. Therefore, effectively improving the production of alkaline proteases remains a research hotspot. In addition, there is no universal medium for alkaline protease production because each strain has its own optimum culture conditions for maximum enzyme production (Adinarayana and Ellaiah 2002). Considering the nonuniformity of culture conditions for improved alkaline protease production, the optimization of fermentation medium constituents and cultural conditions remains one of the most critically investigated phenomena that are carried out before large-scale industrial production.

Response surface methodology (RSM) is a mathematical and statistical model for developing an appropriate functional relationship between a response of interest and multiple variables (Khuri and Mukhopadhyay 2010) and is used as an ideal tool for fermentation process development (Chuprom et al. 2016). The present study aimed to optimize the fermentation medium composition and culture conditions to obtain a higher level of alkaline protease. Single-factor experiments, Plackett‒Burman design and central composite design were applied for optimization of fermentation conditions to improve alkaline protease production by recombinant *B. licheniformis*. The results would be significant for further improving alkaline protease production in the industry.

## Materials and methods

### Strains and culture conditions

*B. licheniformis* BL10 (Wei et al. 2015), with good protein expression capacity, was used as the protein expression host, and the plasmid pHY300PLK was applied to construct a heterologous *aprE* (*B. clausii*) gene expression vector, named pHYaprE. Strain BL10::aprE was plasmid pHYaprE integrated on the chromosome of *B. licheniformis* BL10, which was used to optimize the medium composition, including single-factor experiments, Plackett‒Burman design, steepest-ascent experiment, central composite design and model validation, while strain BL10::aprE/pHYaprE, plasmid pHYaprE free expressed in BL10::aprE, was used to further investigate the generalizability and applicability of the optimized medium. The strains were maintained at − 80 °C in 25% glycerol for long-term storage and activated at 37 °C overnight in solid Luria Bertani (LB) medium.

For seed cultivation, a single colony was picked by a sterile toothpick and transferred into a 50 mL shake flask containing 5 mL of LB medium at 37 °C and 250 rpm for 12 h and then inoculated into a 500 mL shake flask loaded with 50 mL LB at a 3% (v/v) inoculum amount. When strain BL10::aprE/pHYaprE was used, 20 μg/mL tetracycline was added to the LB medium. When the OD_600_ reached 4.0, 1 mL of the seed culture was transferred into a 250 mL shake flask containing 20 mL fermentation medium at 37 °C and 220 rpm.

The initial fermentation medium used for alkaline protease production was composed of the following (g/L): corn starch, 45; soybean meal, 25; yeast extract, 5; (NH_4_)_2_SO_4_, 5; MgSO_4_, 1; and CaCO_3_, 6. The medium was kept at natural pH before sterilization at 121 °C for 20 min. This initial fermentation medium was used to determine the fermentation period and design the single-factor experiment.

### Analytical methods

Cell growth is expressed as colony forming units per millilitre (CFU/mL). Cells were cultured in fermentation medium. The alkaline protease enzyme activity was measured following the Chinese National Standard GB/T23257-2009 Appendix B. One unit of alkaline protease activity (U/mL) was defined as the amount of enzyme that releases 1 μg of tyrosine from casein at 40 °C and pH 10.5.

The total and reducing sugar contents were determined using the 3,5-dinitrosalicylic acid (DNS) assay with modifications (Miller 1959). For the reducing sugars assay, 1 mL of fermentation supernatant with an appropriate dilution ratio was added to a 10 mL colorimetric tube, 1.5 mL of DNS reagent was added, and the mixture was incubated in a boiling water bath for 5 min. After cooling to room temperature, the volume was fixed to 10 mL with deionized water, and the absorbance value was measured at 550 nm. For the total sugar assay, 1 mL of diluted fermentation broth was placed into a 10 mL colorimetric tube, 0.75 mL of 6 M HCl, the tube was placed in a boiling water bath for 20 min and then cooled to room temperature. A total of 1.0 mL 6 M NaOH and 1.5 mL DNS reagent were added to the tube, placed in a boiling water bath for 5 min, and brought to a volume of 10 mL with deionized water, and the absorbance value was measured at 550 nm. A calibration curve was established using glucose as the standard. The Berthelot (Weatherburn 1967) and formaldehyde titration methods (Zou et al. 2009) were used to measure the concentrations of ammonium nitrogen and amino nitrogen, respectively.

### Time course of alkaline protease production in the initial medium

The time course of growth and alkaline protease production was studied by culturing strain BL10::aprE in the initial fermentation medium. 20 mL of the sterile initial fermentation medium in 250 ml flask was inoculated with strain BL::aprE. The shake flasks were incubated at 37 °C and 220 rpm for 84 h, and the protease activity was determined every 12 h during fermentation to determine the fermentation period.

### Single-factor experiments

In single-factor experiments, only one factor or variable affecting protease production was varied at a time while keeping other variables constant (Vineeta et al. 2016). In this study for exploration the approximate concentration range of each component of the fermentation medium, only one of the parameters in the initial fermentation medium was varied at a time by keeping all other factors constant. To study the influence of carbon source concentration on alkaline protease production, different corn starch concentrations from 30 g/L to 105 g/L were selected to optimize the medium condition. The effect of nitrogen source on protease production was evaluated using different concentrations of soybean meal (10, 25, 40, 55, and 70 g/L), yeast extract (1, 3, 5, 7, and 9 g/L), and (NH_4_)_2_SO_4_ (1, 2, 3, 4, and 5 g/L). Various concentrations of MgSO_4_ (0.5, 1, 2, 3, and 4 g/L) were used to examine their effects on alkaline protease production. To choose the best initial pH of the medium before sterilization and inoculation amount, pH values from 6.0 to 10.0 and inoculation amounts between 1% (v/v) and 5% (v/v) were tested in inoculated flasks. The pH was adjusted with 1 N HCl or 1 M NaOH before sterilization and pH determination of the medium before and after sterilization with a pH meter. All experiments were carried out in triplicate.

### Plackett‒Burman design

The Plackett‒Burman design has been used to screen the major components of the medium for their separate major effects based on the results of single-factor experiments (Elsanhoty et al. 2012). Seven different nutritional and environmental factors, including corn starch, soybean meal, yeast extract, (NH_4_)_2_SO_4_, MgSO_4_, initial medium pH value before sterilization, inoculation amount, and four dummy factors, were used to measure the standard error of the design. A 15-run design with three center points and twelve cube points on two levels (+ 1, − 1) was implemented using Design-Export Software 13.0. The design of Plackett‒Burman is shown in Additional file 1: Table S1. The influence of each factor on alkaline protease production was described by the first-degree mode:

$$y={\beta }_{0}+\sum {\beta }_{i}{X}_{i}$$

where *Y* is the predicted response for alkaline protease activity (the response), β_0_ is the model intercept term, *β*_i_ is the linear coefficient, and *X*_i_ is an independent variable (Tsigoriyna et al. 2021). All experiments were conducted in triplicate.

### Steepest-ascent method

After the screening of the significant factors, the next step was to confirm the optimum values of these factors. To determine whether these components fall within the desirable range, the significance between the central point and the average output response of the Plackett‒Burman design was examined. If there was no significance, the steepest-ascent experiment was used to optimize the conditions and get as close to the optimal area as possible (Iqbal et al. 2016). The steepest-ascent approach was a useful strategy used in RSM optimization experiment to move identified significant factors of Plackett‒Burman design in current operating condition close to the optimum region (Ekpenyong et al. 2021). And based on the above regression analysis of the Plackett‒Burman design, appropriate direction and step length of significant factors were determined by the steepest-ascent method (Fan et al. 2020). A particular point where the alkaline protease production was highest by the steepest ascent design would be near the optimal point and could be used as the center point of the response surface.

### Central composite design (CCD)

As a second-degree model, CCD is perhaps the most popular model in classical RSM. In this work, CCD with three factors (corn starch content, soybean meal content, and initial medium pH value) at three levels (-1,0,1) was used to evaluate the effect of interactions between these three factors. A total of 19 randomized experiments consisting of five replications at the center point were employed by Design-Export Software 13.0. The design of the CCD is shown in Additional file 1: Table S2. The second-degree mode used to analyse the experimental data is shown as follows:

$$y={\beta }_{0}+\sum {\beta }_{i}{X}_{i}+\sum {\beta }_{ii}{X}_{i}^{2}+\sum {\beta }_{ij}{X}_{i}{X}_{j}$$

where *Y* is the predicted response for alkaline protease activity, β_0_ is the model intercept term, *β*_i_ is the linear coefficient, and *X*_i_ and *X*_j_ are independent variables (Maity and Mishra 2019). Each experiment was performed in triplicate.

### Verification and evaluation of the software optimization medium

The validation of the model equation was carried out in triplicate for the production of alkaline protease under optimal conditions, and the results obtained empirically were compared with the response predicted by the model. In total, 27 shake flasks with 20 mL working volume for each were incubated at at 37 °C and 220 rpm for 90 h. In addition to protease, fermentation process parameters, including cell growth, reducing and total sugars, pH, ammonium nitrogen, and amino nitrogen, were also measured in the optimized medium. Take samples at different time intervals for analysis.

### Further investigation of the applicability of the optimized medium

To promote the universality and applicability of the optimized culture medium, we used a strain BL::aprE/pHYaprE with high expression of alkaline protease to test whether the optimized culture medium can further improve the production of protease. The experiments were performed three times.

## Results

### Time course of alkaline protease production in the initial medium

**Fig. 1:**
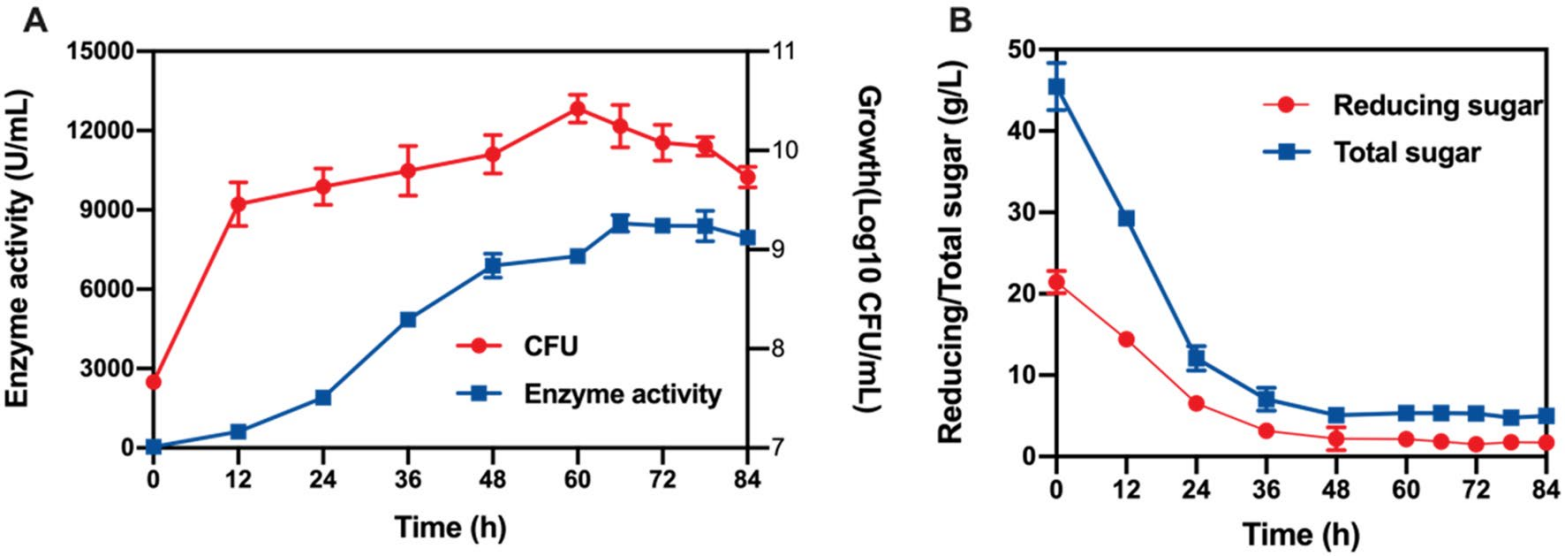
The process of alkaline protease production using the initial fermentation medium

To test the enzyme production capacity of the initial fermentation medium, product accumulation, cell growth and sugar consumption during fermentation were measured at the shake flask level (Fig. 1). The number of viable cells in the culture started immediately after inoculation and peaked at 60 h. Alkaline protease was produced as the biomass accumulated, reaching a maximum level of 8491.23 U/mL after 66 h of fermentation, while the concentrations of reducing and total sugar declined sharply at the beginning of fermentation and thereafter remained at extremely low levels after 48 h. Based on the above results, 66 h of fermentation was chosen as the fermentation cycle for the subsequent experiments.

### Single-factor experiments

#### Effect of nitrogen source concentrations

**Fig. 2:**
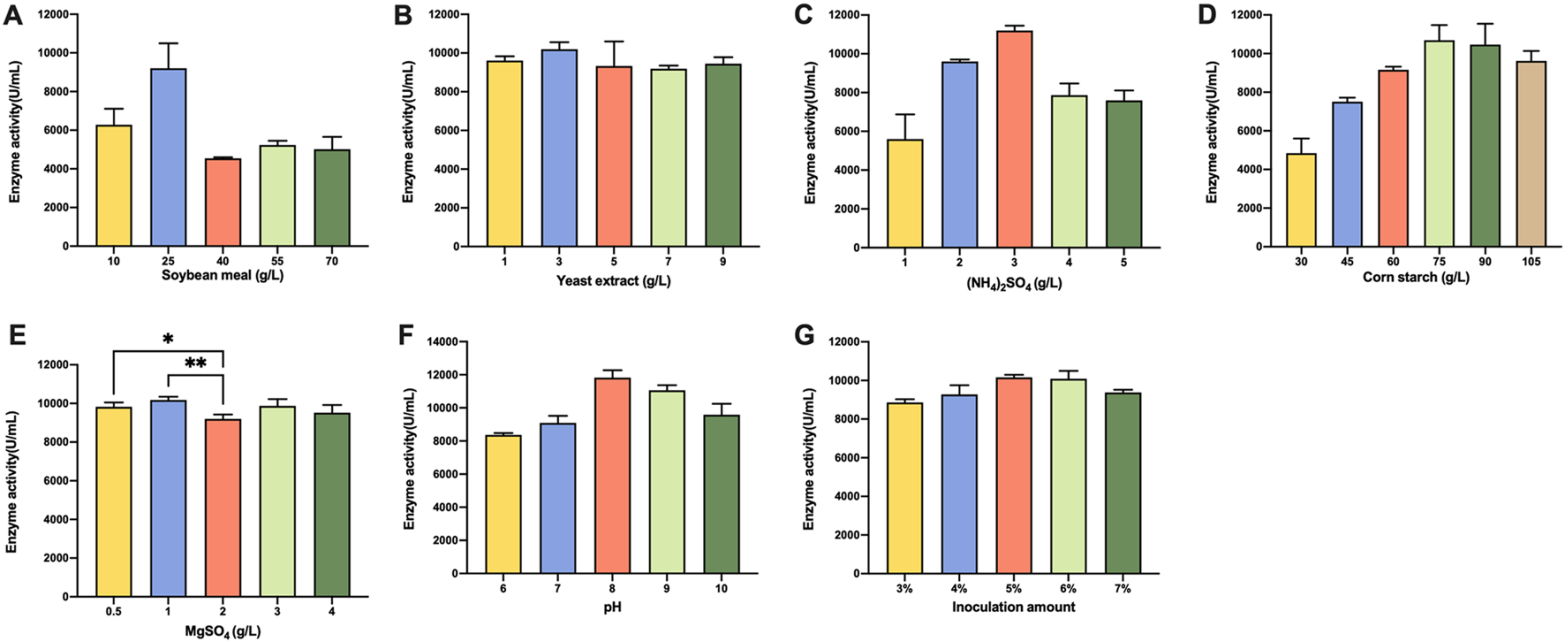
Effect of different nitrogen sources on alkaline protease production

The kind of nitrogen source and its concentration in the culture are key factors in the synthesis of proteases. Nitrogen sources can be divided into effective nitrogen sources and slow-release nitrogen sources, and rational usage can effectively regulate the growth rate of bacteria and the synthesis of target products. To study the effect of slow-release nitrogen sources (soybean meal) and effective nitrogen sources (yeast extract and (NH_4_)_2_SO_4_), a series of experiments were carried out. The results demonstrated that the maximal yield of protease production was obtained under 25 g/L soybean meal (Fig. 2A), 3 g/L yeast extract (Fig. 2B), and 3 g/L (NH_4_)_2_SO_4_ (Fig. 2C).

#### Effect of carbon source concentration

Carbon is the most crucial medium component since it serves as a source of energy for microorganisms and plays an important role in growth as well as in the production of primary and secondary metabolites. The production of alkaline proteases by *B. licheniformis* DW2 was examined using various amounts of corn starch, which is regarded as a low-cost carbon source for industrial manufacturing. The results showed that raising the concentration of corn starch to 75 g/L increased the enzymatic activity, and then a decrease in the alkaline protease activity continued gradually as the concentration of corn starch increased, indicating that 75 g/L corn starch was favourable for the strain to produce protease (Fig. 2D).

#### Effect of MgSO_4_ concentration

Metal cations have a great effect on improving the activity and stability of proteases. Mg^+^ is an activator of various enzymes that are widely involved in material metabolism and energy metabolism. Therefore, the effect of 0.5 g/L, 1 g/L, 2 g/L, 3 g/L, and 4 g/L MgSO_4_ on protease production was studied, and the results showed that the concentration of MgSO4 at 1 g/L corresponded to slightly higher alkaline protease enzyme activity than the other groups (Fig. 2E).

#### Effect of initial pH value

The pH of the fermentation medium has an important effect on the enzymatic reaction and the charge and membrane permeability of the bacterial cell membrane. Since it is challenging to regulate pH during shake flask fermentation, this experiment focused on how the medium's initial pH affected the protease activity. Five pH values ranging from 6.0 to 10.0 were tested. The data indicated that the increase in initial medium pH (6.0–8.0) resulted in an obvious improvement in protease production, followed by a slight decrease at pH 9.0. Consequently, pH 8.5 was chosen as the condition for subsequent optimization (Fig. 2F).

#### Effect of inoculum amount

An optimum initial inoculum amount was essential for protease production. If the inoculum amount is too small, an insufficient number of bacteria would prolong the fermentation period and reduce the yield of the target product; conversely, too large of an inoculum amount would also cause scant dissolved oxygen and affect the synthesis of the target product. Finding the optimal inoculum size is important in the fermentation process. In this experiment, the inoculum amount was set to 3%, 4%, 5%, 6% and 7%. The results of shaking flask fermentation are shown in Fig. 2G. The protease enzyme activity increased with increasing inoculum amount and reached a maximum at 5%. When the inoculation amount continued to increase, the protease enzyme activity showed a downwards trend. One of the possible reasons for this observation was that the dissolved oxygen was insufficient under high inoculum amounts.

The above single-factor experiments revealed that 25 g/L soybean meal, 3 g/L yeast extract, 3 g/L (NH_4_)_2_SO_4_, 75 g/L corn start, 1 g/L MgSO_4_, pH 8.5, and 5% inoculum amount were the best conditions for BL10::aprE to produce alkaline protease. Thus, these conditions were selected as the optimization steps for the following experiment.

### Plackett–Burman design

According to the Plackett‒Burman design, fifteen experiments, each with its characteristic combination of the tested factors, were performed, and the response values were calculated and are shown in Additional file 1: Table S3. The data described in Additional file 1: Table S3 revealed that the alkaline protease activity in 15 trials ranged considerably from 6560.24 to 11,279.016 U/mL. On the basis of the data obtained by this design, a regression equation was generated to establish the correlation between the independent factors and the dependent factor. The model was expressed as follows:

$$Y=8257.85+863.99\mathrm{A}+529.90\mathrm{B}-197.41\mathrm{C}+66.34\mathrm{D}+322.10\mathrm{E}+804.85\mathrm{F}-72.73\mathrm{G}-486.74\mathrm{H}+120.69\mathrm{J}-227.79\mathrm{K}-125.48\mathrm{L}$$

In addition, the significance of independent factors influencing alkaline protease production by BL10::aprE is summarized in Additional file 1: Table S4. Within the prescribed ranges, corn starch content, soybean meal content, and initial medium pH had significant effects on alkaline protease production (*p*-values less than 0.05), while other components with *p*-values greater than 0.05 were determined nonsignificant. The coefficient of determination *R*^2^ (0.9725) indicated that the model could fit approximately 97% of the experimental data, and the model's *p*-value was less than 0.05, implying that the model was significant. The results of the *t* test for variance between the observed average of this model and the center point experiments showed that the difference was not significant, indicating that the optimum point was not in the scope of our experiment. Experimentation on the steepest ascent path was necessary to reach the optimal region.

### Steepest ascent experiment

The result of the Plackett‒Burman design indicated that the optimal region was outside the current design space. Therefore, the steepest ascent method was carried out to determine the center points of the significant factors. Based on the regression equation of the Plackett‒Burman design, the path of the steepest ascent was aimed at increasing the levels of corn starch and soybean meal and pH of the initial medium to improve the production of alkaline protease. The design and results of the steepest ascent experiment are listed in Additional file 1: Table S5. The maximum production of the alkaline protease was achieved in run 3 (12,938.26 U/mL), and after the third step on the path, further experimentation could not increase the protease activity. These data showed that the results were approaching the neighbourhood of the optimum region. The composition of the third set of experiments was chosen for the center point of the CCD design.

### Central composite design

After identifying the optimal region, a CCD experiment was used to further optimize the production of alkaline protease. The randomized design matrix and the analysis of the factors are shown in Additional file 1: Tables S6 and S7, respectively. Using Design-Export 13.0 to perform quadratic polynomial regression fitting on the CCD test results, the regression equation obtained was:

$$Y=15064.95-372.27A+137.47\mathrm{B}- 65.44\mathrm{C }+ 223.03\mathrm{AB }- 326.06\mathrm{AC }+ 776.91\mathrm{BC }- 721.44{\mathrm{A}}^{2}- 645.31{B}^{2} - 440.26{\mathrm{C}}^{2}.$$

The analysis of variance revealed that the response surface regression model can explain 80.86% of the variation in the data (*R*^2^ = 0.8086), indicating that the model had a good fitting degree (values of *R*^2^ > 0.75 indicate good model fitness). The *P*-value of the model (*P* = 0.0216, *P* < 0.05) and “Lack-of-Fit” value (*P* = 0.1642,* P* > 0.05) showed that the proportion of nonnormal errors between the equation and the actual fitting was small, and this model can better predict the optimal conditions for producing alkaline protease. In addition, the input variables and their interaction effects are shown in Additional file 1: TableS7. The *P*-values of models BC, A^2^, and B^2^ are < 0.05, indicating that the interaction of BC and the influence of Factors A^2^ and B^2^ on the response value are relatively significant. The *P*-values of A^2^ and B^2^ were ≤ 0.01, indicating that these two factors had an extremely significant impact on the response value, while the other terms had no significant effect on the response value.

**Fig. 3:**
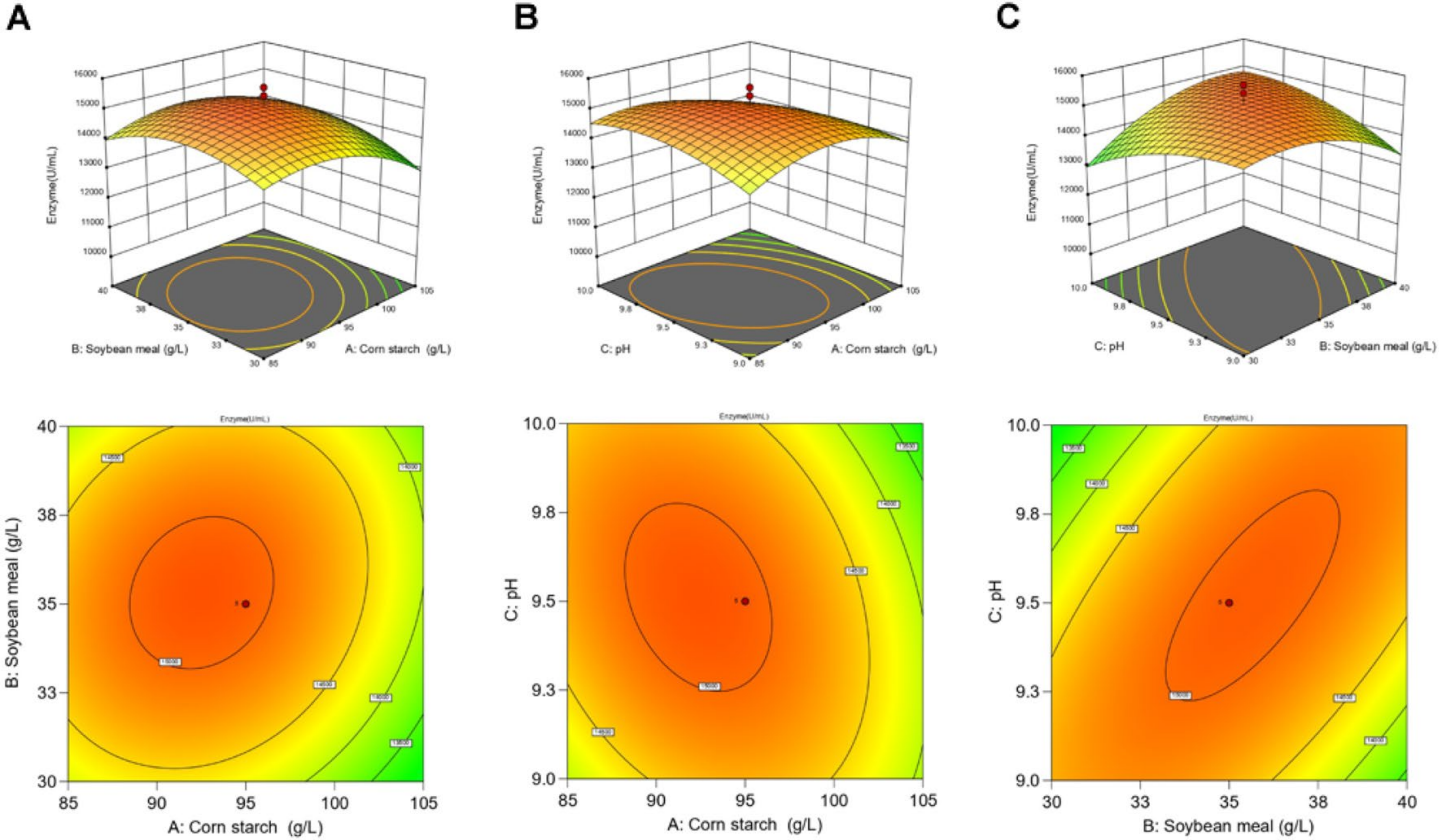
Response surfaces and contour plots describing the interaction of different variables on alkaline protease production. Variables are soybean meal and corn starch contents (**A**), corn starch content and pH (**B**), and soybean meal content and pH (**C**)

The three-dimensional response surface and two-dimensional contour plot were applied to describe the interaction of different factors and the effect of each factor change on alkaline protease production. According to the regression equation fitted above, Design-Expert 13.0 software was used to draw the response surface curve and contour plots by changing two variables within the experimental range, while the other variable remained at zero level, as shown in Fig. 3. In the response surface diagram, the steeper the curve trend is, the more significant the influence of the research factors on the results. The elliptic contour plot indicates that the interaction between research factors is significant, while the circular contour plot indicates that the interaction is weak.

Figure 3A shows the relationship between corn starch and soybean meal contents at an initial medium pH of 9.5. The highest predicted activity of alkaline protease was 15,115.5 U/mL when the levels of corn starch and soybean meal were adjusted to 92.52 and 35.34 g/L, respectively. The resulting 3D surface plot showed that enzyme activity was enhanced by increasing the concentrations of corn starch and soybean meal, and further increasing the concentration of these two components resulted in a decrease in the activity of alkaline protease, which may be due to insufficient dissolved oxygen in the culture medium. Figure 3B presents the outcome of the interaction between corn starch concentration and initial medium pH. The highest predicted activity of alkaline protease was 15,113.2 U/mL when the corn starch content was 92.39 g/L and the pH was 9.51. The enzyme activity of the target product alkaline protease showed a gradual increase with increasing pH, while with increasing corn starch concentration, enzyme activity increased first and then tended to decrease, indicating that the effect of corn starch on enzyme activity was more significant than pH, which was also consistent with the results in Additional file 1: Table S7 that the* p*-value of corn starch was smaller than that of pH. The effect of soybean meal concentration and initial medium pH at fixed values of corn starch concentration is illustrated in Fig. 3C, which shows that the predicted maximum enzyme activity was 15,072.6 U/mL when the soybean meal concentration was 35.64 g/L and the pH was 9.52. The degree of ellipticity of the contour plot in Fig. 3C was the largest than Fig. 3A , B, indicating that the interaction between soybean meal and initial medium pH was the most obvious. This was also supported by the ANOVA results in Additional file 1: Table S7, where the *p*-value for BC was lower than those for AB and AC.

### Verification and evaluation of the software optimization medium

**Fig. 4:**
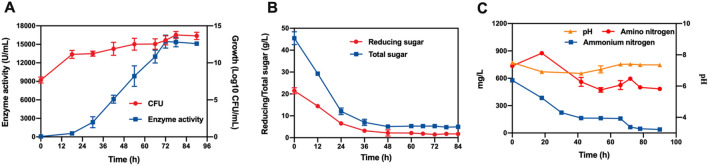
Time course of fermentation in the optimum culture medium. Changes in alkaline protease activity and biomass (**A**), reducing and total sugar levels (**B**), ammonium nitrogen, amino nitrogen concentration and fermentation process pH (**C**)

The maximal protease activity predicted by the model obtained from CCD was 15,120.97 U/mL. To verify the reliability of the model, the efficiency of the optimized medium was assessed in shake flask fermentation using the strain recombinant *B. licheniformis* BL10::aprE, and the observed protease activity was 15,435.1 U/mL (Fig. 4), which deviated from the predicted value by 2%, indicating that the model was able to predict the actual protease enzyme activity well. We also explored the fermentation process in the optimized medium, and the results are shown in Fig. 4. It seemed that the microbial growth and the synthesis of the product were correlated, and the number of viable cells in the culture and enzyme activity tended to increase in the first 66 h of fermentation. The maximum alkaline protease activity was obtained at 72 h of fermentation and remained steady for approximately 12 h, greater than the initial fermentation medium enzyme activity by 82%. Total sugars, reducing sugars, ammonium nitrogen, amino nitrogen and pH are important process indicators for the condition of microbial metabolism. As shown in Fig. 4, the pH decreased progressively at first with decreasing ammonium nitrogen, reducing sugar and total sugar levels, and then the pH was gradually restored. Changes in pH can be attributed to substrate consumption and ammonium production, particularly in the case of amino acids. In the early stages of fermentation, the bacteria absorb soybean meal to release amino nitrogen, which is then gradually consumed with the synthesis of protease until a low level is maintained. As a result, optimizing the alkaline protease fermentation medium using CCD response surface methodology is practicable, effective, and practical.

### Further investigation of the applicability of the optimized medium

**Fig. 5:**
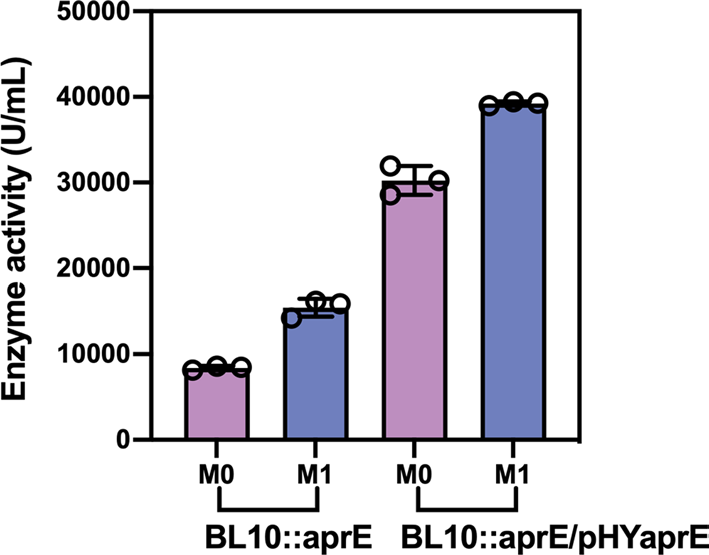
Alkaline protease synthesis in different media (M0: initial medium; M1: optimized medium) and strains (BL10::aprE and BL10::aprE/pHYaprE)

To further test the generalizability and applicability of the optimized medium, we performed shake flask fermentation by an overexpressed strain BL10::aprE/pHYaprE in optimized medium and initial medium. As shown in Fig. 5, when cultivated in the initial fermentation medium, the enzyme activity of BL10::aprE/pHYaprE was 30,230.8 U/mL. Using the optimized culture medium, enzyme activity reached a maximum of 39,233.6 U/mL, greater than before optimization by 28.8%, indicating that the developed medium also has good applicability in this high-yielding strain.

## Discussion

Alkaline proteases have a wide range of commercial applications, which has piqued the interest of both industry and academia. *B. licheniformis* is considered to be a relatively good protein-producing bacterium compared to all the protease-producing bacteria, and it can secrete large quantities of proteins, up to 20–25 g/L (Veith et al. 2004; Voigt et al. 2009). The strain's maximum productivity is dependent on a suitable medium and culture conditions. For the manufacture of alkaline protease enzyme by *B. licheniformis* NCIM-2042, Biswanath et al. (2010) selected optimal carbon, nitrogen, and sulfate sources using a one-variable-at-a-time strategy, and the maximum yield of this enzyme increased nearly fourfold. *B. licheniformis* MZK05M9 alkaline protease production was adjusted by Mian et al. (2018) using CCD, and the maximum alkaline protease production was 560 U/mL, which showed an overall 36.6% enhancement over the basal medium. To fully comprehend and optimize the culture medium, we combined conventional "single-factor experiment" techniques with statistical methods, which allowed us to determine the optimal conditions and led to a protease activity of 15,435.1 U/mL, 82% greater than that of the initial medium. Our results showed that the yield of alkaline protease from *B. clausii* was higher than that of alkaline protease optimized by others under the same method (Kumar et al. 2014; Oskouie et al. 2008).

For industrial production, the use of low-cost medium can significantly reduce fermentation costs. The use of various agricultural residues to produce proteases from *Bacillus*, including shells, wheat grains and soybean meal, has been extensively described in the literature (Biswanath et al. 2012). In our culture medium, inexpensive and abundantly available soybean meal was chosen as the primary organic nitrogen source. Since soybean meal is insoluble, an excess amount could lower the dissolved oxygen needed for fermentation. We found that the content of soybean meal in the optimized culture medium was lower than that in the initial medium, indicating that the reduction in soybean meal somewhat increased the dissolved oxygen in the culture medium to a certain extent, which was more conducive to cell growth. Similar findings have been reported by other researchers (Hammami et al. 2018). Soybean meal often takes the form of a macromolecular protein that can be gradually broken down by the protease released by cells, making it difficult for cells to grow in the early stages of fermentation. Therefore, we added a small amount of yeast extract and ammonium sulfate as quick-acting nitrogen sources to achieve the goal of increasing production by taking advantage of the coordination between the cell growth period and the formation period of metabolic products. Zambare et al. (2011) also found that maximum protease production was obtained with soybean meal and tryptone and that using either would dramatically lower the enzyme activity.

Many enzymatic reactions and the transport of various components across the cell membrane are affected by the pH of the culture. We noticed that the optimal initial pH of the medium was relatively alkaline for the synthesis of proteases, a discovery that is consistent with that described by Hakim et al. (2018). Although the initial pH of the medium was adjusted to alkaline when preparing the medium, after sterilization, the pH level usually declined to approximately 7.5. Therefore, we investigated the fermentation processes in various initial pH values of the medium before sterilization and discovered that it grew with the increase in the medium pH before sterilization and that the enzyme activity also increased (Additional file 1: Fig. S1). In general, the pH value of the medium decreased after autoclaving, creating differences in cell growth and product synthesis, which might be caused by significant changes in the composition of the medium after autoclaving. We found that during the whole fermentation process, the pH displayed a trend of decreasing and then increasing until the starting pH was restored (Fig. 4B). The pH change in the fermentation broth is a comprehensive result of the metabolic reaction of the bacterium, while the mechanism of the internal response of the bacterium to the pH change needs to be further explored.

Protein engineering and genetic engineering are also useful strategies for improving strain creation. As a secreted protein, a suitable signal peptide would have a positive impact on enhancing the expression levels of alkaline protease and secretion efficiency. The effect of several signal peptides on BcaPRO secretion was studied by Liu et al. (2019), who discovered that using SP_DacB_ enhanced BcaPRO enzyme activity 2.9 fold. To show that increasing gene copy number can successfully increase the expression level of protease, Zhou et al. (2021) added two additional copy number expression frames on the chromosome, which increased protease synthesis by 136%. In this work, we selected the alkaline protease gene containing its own signal peptide from *B. clausii* and expressed it in single-copy form in the *B. licheniformis* BL10 strain using the optimal medium to achieve an enzyme activity of 15,435.1 U/mL. After overexpressing the *aprE* gene by episomal plasmid, the enzyme activity further increased by 154% to reach 39,233.6 U/mL. Therefore, the strain employed in this investigation has a high potential for productivity modification, and we reasoned that an adequate signal peptide or gene expression dose would be responsible for further improving the synthesis of protease. Despite considerable progress in enhancing enzyme activity by medium modification and genetic and protein engineering technologies, the mechanism of enzyme-producing metabolism within *B. licheniformis* cells remains unknown. Genome-scale metabolic network models are widely used to resolve complex metabolic networks and endogenous metabolite synthesis mechanisms in microorganisms (Kerkhoven 2021; Loghmani et al. 2022). Therefore, work is underway to use a genomic network metabolic model to better understand the cellular response to the environment and enzyme production mechanisms, with the goal of purposefully guiding the design of metabolic engineering strategies to achieve alkaline protease production based on medium optimization for industrial applications.

## Conclusion

Alkaline protease is a major industrial enzyme that is widely used in detergent, food, and waste management. This work used a preliminary exploration of the fermentation process to pinpoint the cultural endpoint. Then, the culture conditions and medium components were optimized using traditional methods and statistical tools to develop a medium that supported the stable production of alkaline protease. When compared to the unoptimized fermentation broth, the enzymatic activity of alkaline protease in the optimized broth increased by approximately 82% from 8491.23 U/mL to 15,435.1 U/mL using strain BL10::aprE. Moreover, an overexpression strain BL10::aprE/pHYaprE was used to further investigate the application of the optimized medium, and the production of alkaline protease increased by approximately 30% from 30,230.8 to 39,233.6 U/mL, which was the highest level ever recorded in shaking flasks. This study provides a theoretical basis and technical support for further optimization of the protease fermentation process and industrial production applications.

### Supplementary Information

**Additional file 1: Table S1.** Factors and levels of Plackett-Burman experiment. **Table S2.** Factors and levels of Central Composite design. **Table S3.** Plackett-Burma test design and results. **Table S4.** The variance analysis of Plackett-Burman test. **Table S5.** The design and results of steepest ascent experiment. **Table S6.** CCD design and results. **Table S7.** The variance analysis of experiment. **Figure S1.** The alkaline protease and process pH during the fermentation of different initial medium pH.

## Data Availability

The data supporting the conclusions are included in the main manuscript and its additional files.
