# Supplementary material for: Enhancement of alkaline protease production in recombinant Bacillus licheniformis by response surface methodology
Source: Bioresour Bioprocess. 2023 Apr 20;10(1):27. doi: 10.1186/s40643-023-00641-8 (PMC10991860; doi:10.1186/s40643-023-00641-8)

**Supplementary Information**

**Table S1** Factors and levels of Plackett-Burman experiment

| Variables | | Experimental Levels | | | |
| --- | --- | --- | --- | --- | --- |
| Number | Variables | | -1 | 0 | 1 |
| A | Corn starch | | 56.25 | 75 | 93.75 |
| B | Soybean meal | | 18.75 | 25 | 31.25 |
| C | Yeast extract | | 2.25 | 3 | 3.75 |
| D | (NH_4_)_2_SO_4_ | | 2.25 | 3 | 3.75 |
| E | MgSO_4_ | | 0.75 | 1 | 1.25 |
| F | pH | | 7.5 | 8 | 9.5 |
| G | Inoculation amount | | 3.75 | 5 | 6.25 |
| H | Dummy1 | | -1 | 0 | 1 |
| J | Dummy2 | | -1 | 0 | 1 |
| K | Dummy3 | | -1 | 0 | 1 |
| L | Dummy4 | | -1 | 0 | 1 |

**Table S2** Factors and levels of Central Composite design

| Factors | |  | Experimental Levels | |
| --- | --- | --- | --- | --- |
| number | Factors | -1 | 0 | 1 |
| A | Corn starch(g/L) | 85 | 95 | 105 |
| B | Soybean meal(g/L) | 30 | 35 | 40 |
| C | pH | 9 | 9.5 | 10 |

**Table S3** Plackett-Burma test design and results

| Random number | Run number | Independent variable | | | | | | | | | | | | Response  U/mL |
| --- | --- | --- | --- | --- | --- | --- | --- | --- | --- | --- | --- | --- | --- | --- |
|  |  | A | B | C | D | E | F | G | H | J | K | L |  | |
| 15 | 1 | 0 | 0 | 0 | 0 | 0 | 0 | 0 | 0 | 0 | 0 | 0 | 8938.81 | |
| 5 | 2 | -1 | -1 | 1 | -1 | 1 | 1 | -1 | 1 | 1 | 1 | -1 | 7231.61 | |
| 3 | 3 | 1 | -1 | 1 | 1 | -1 | 1 | 1 | 1 | -1 | -1 | -1 | 8516.81 | |
| 11 | 4 | 1 | -1 | 1 | 1 | 1 | -1 | -1 | -1 | 1 | -1 | 1 | 8660.67 | |
| 10 | 5 | -1 | 1 | 1 | 1 | -1 | -1 | -1 | 1 | -1 | 1 | 1 | 5677.87 | |
| 2 | 6 | -1 | 1 | 1 | -1 | 1 | 1 | 1 | -1 | -1 | -1 | 1 | 9082.68 | |
| 14 | 7 | 0 | 0 | 0 | 0 | 0 | 0 | 0 | 0 | 0 | 0 | 0 | 8440.08 | |
| 6 | 8 | -1 | -1 | -1 | 1 | -1 | 1 | 1 | -1 | 1 | 1 | 1 | 7691.98 | |
| 9 | 9 | 1 | 1 | 1 | -1 | -1 | -1 | 1 | -1 | 1 | 1 | -1 | 8593.54 | |
| 4 | 10 | -1 | 1 | -1 | 1 | 1 | -1 | 1 | 1 | 1 | -1 | -1 | 7519.34 | |
| 13 | 11 | 0 | 0 | 0 | 0 | 0 | 0 | 0 | 0 | 0 | 0 | 0 | 8593.54 | |
| 8 | 12 | 1 | 1 | -1 | -1 | -1 | 1 | -1 | 1 | 1 | -1 | 1 | 9974.64 | |
| 1 | 13 | 1 | 1 | -1 | 1 | 1 | 1 | -1 | -1 | -1 | 1 | -1 | 11279.016 | |
| 12 | 14 | -1 | -1 | -1 | -1 | -1 | -1 | -1 | -1 | -1 | -1 | -1 | 6560.24 | |
| 7 | 15 | 1 | -1 | -1 | -1 | 1 | -1 | 1 | 1 | -1 | 1 | 1 | 7106.93 | |

**Table S4** The variance analysis of Plackett-Burman test

| Source | Sum of Squares | Degree of freedom | Mean square | *F* | *P* |  |
| --- | --- | --- | --- | --- | --- | --- |
| Model | 2.576E+007 | 11 | 2.342E+006 | 9.63 | 0.0439 | significant |
| A | 8.958E+006 | 1 | 8.958E+006 | 36.84 | 0.0090 | * |
| B | 3.370E+006 | 1 | 3.370E+006 | 13.86 | 0.0337 | * |
| C | 4.667E+005 | 1 | 4.667E+005 | 1.92 | 0.2595 |  |
| D | 52809.16 | 1 | 52809.16 | 0.22 | 0.6729 |  |
| E | 1.245E+006 | 1 | 1.245E+006 | 5.12 | 0.1086 |  |
| F | 7.773E+006 | 1 | 7.773E+006 | 31.97 | 0.0110 | * |
| G | 63479.18 | 1 | 63479.18 | 0.26 | 0.6446 |  |
| H | 2.843E+006 | 1 | 2.843E+006 | 11.69 | 0.0419 | * |
| J | 1.748E+005 | 1 | 1.748E+005 | 0.72 | 0.4588 |  |
| K | 6.226E+005 | 1 | 6.226E+005 | 2.56 | 0.2078 |  |
| L | 1.890E+005 | 1 | 1.800E+005 | 0.78 | 0.4429 |  |
| Residual | 7.294E+005 | 3 | 2.431E+005 |  |  |  |
| Lack of Fit | 5.989E+005 | 1 | 5.989E+005 | 9.18 | 0.0939 |  |
| Pure Error | 1.305E+005 | 2 | 65249.64 |  |  |  |
| Cor Total | 2.649E+007 | 14 |  |  |  |  |

*F*: Fishers’s function; *P*: Level of significance; *R*^2^ = 0.9725

∗Significant at 95% confidence level.

**Table S5** The design and results of steepest ascent experiment

| Number | Corn starch  （g/L） | Soybean meal（g/L） | pH | Activity（U/mL） |
| --- | --- | --- | --- | --- |
| 1 | 75 | 25 | 8.5 | 9945.87 |
| 2 | 85 | 30 | 9 | 10300.73 |
| 3 | 95 | 35 | 9.5 | 12938.26 |
| 4 | 105 | 40 | 10 | 9619.77 |
| 5 | 115 | 45 | 10.5 | 6445.15 |

**Table S6** CCD design and results

| Random number | Run number | Factors | | | Response value  U/mL |
| --- | --- | --- | --- | --- | --- |
|  |  | A | B | C |  |
| 2 | 1 | 1 | -1 | -1 | 14290.59 |
| 3 | 2 | -1 | 1 | -1 | 12756.03 |
| 19 | 3 | 0 | 0 | 0 | 14472.82 |
| 17 | 4 | 0 | 0 | 0 | 15710.06 |
| 6 | 5 | 1 | -1 | 1 | 10761.10 |
| 7 | 6 | -1 | 1 | 1 | 13638.40 |
| 10 | 7 | 1.682 | -1 | 0 | 12756.03 |
| 15 | 8 | 0 | -1 | 0 | 14721.41 |
| 4 | 9 | 1 | 1 | -1 | 12007.93 |
| 18 | 10 | 0 | 0 | 0 | 15441.51 |
| 9 | 11 | -1.682 | 0 | 0 | 13783.22 |
| 1 | 12 | -1 | -1 | -1 | 13916.54 |
| 13 | 13 | 0 | 0 | -1.682 | 13954.31 |
| 16 | 14 | 0 | 0 | 0 | 14894.82 |
| 12 | 15 | 0 | 1.682 | 0 | 14242.64 |
| 5 | 16 | -1 | -1 | 1 | 13705.54 |
| 14 | 17 | 0 | 0 | 1.682 | 14175.50 |
| 11 | 18 | 0 | -1.682 | 0 | 12727.26 |
| 8 | 19 | 1 | 1 | 1 | 13600.32 |

**Table S7** The variance analysis of experiment

| Source | Sum of Squares | Degree of freedom | Mean square | *F* | *P* |
| --- | --- | --- | --- | --- | --- |
| Model | 2.045E+007 | 9 | 2.272E+006 | 4.22 | 0.0216 |
| A | 1.893E+006 | 1 | 1.893E+006 | 3.52 | 0.0934 |
| B | 2.581E+006 | 1 | 2.581E+006 | 0.48 | 0.5060 |
| C | 58489.50 | 1 | 58489.50 | 0.11 | 0.7491 |
| A*B | 3.979E+005 | 1 | 3.979E+005 | 0.74 | 0.4121 |
| A*C | 8.505E+005 | 1 | 8.505E+005 | 1.58 | 0.2402 |
| B*C | 4.829E+006 | 1 | 4.829E+006 | 8.98 | 0.0151 |
| A^2^ | 7.105E+006 | 1 | 7.105E+006 | 13.21 | 0.0054 |
| B^2^ | 5.684E+006 | 1 | 5.684E+006 | 10.57 | 0.0100 |
| C^2^ | 2.646E+006 | 1 | 2.646E+006 | 4.92 | 0.0536 |
| Residual | 4.841E+006 | 9 | 5.397E+005 |  |  |
| Lack of Fit | 3.787E+006 | 5 | 7.574E+005 | 2.87 | 0.1642 |
| Pure Error | 1.054E+006 | 4 | 2.635E+005 |  |  |
| Cor Total | 2.529E+007 | 18 |  |  |  |

*F*: Fishers’s function; *P*: Level of significance; *R*^2^ = 0.8086

**Figure S1** The alkaline protease and process pH during the fermentation of different initial medium pH


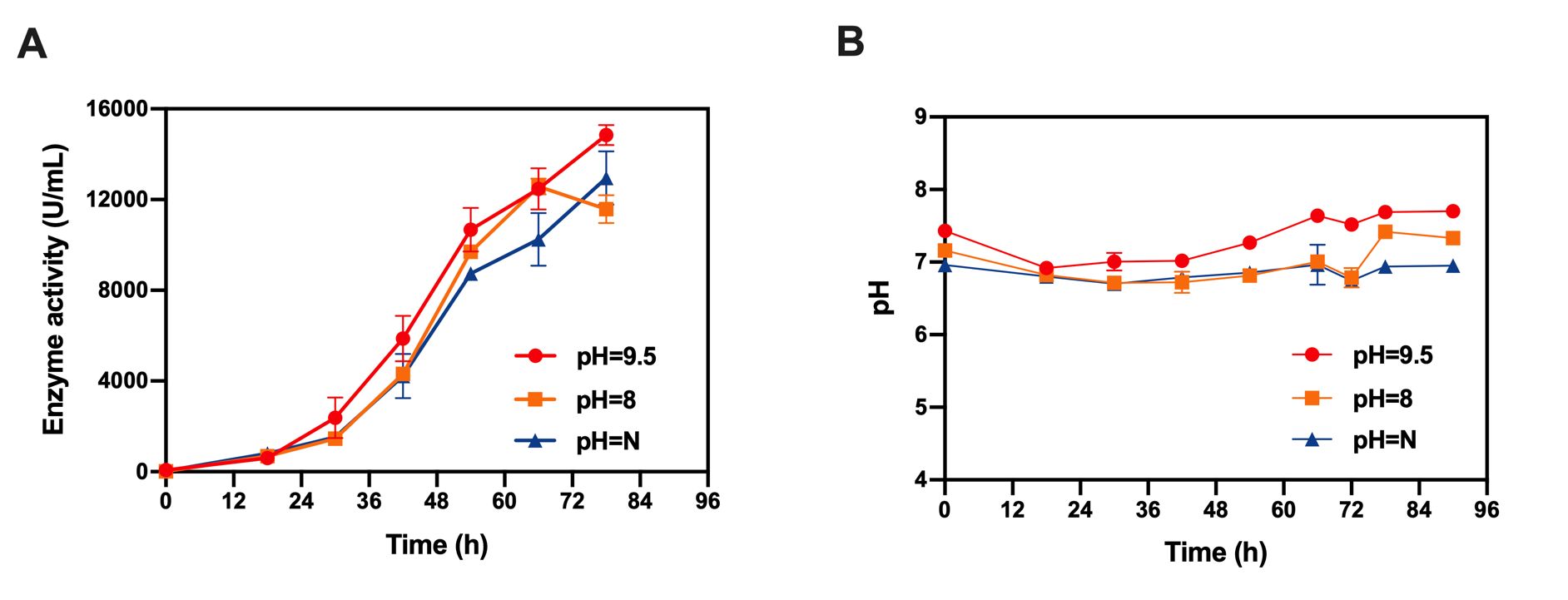

Supplement: Supplementary file 1 — Additional file 1: Table S1. Factors and levels of Plackett-Burman experiment. Table S2. Factors and levels of Central Composite design. Table S3. Plackett-Burma test design and results. Table S4. The variance analysis of Plackett-Burman test. Table S5. The design and results of steepest ascent experiment. Table S6. CCD design and results. Table S7. The variance analysis of experiment. Figure S1. The alkaline protease and process pH during the fermentation of different initial medium pH. [file 40643_2023_641_MOESM1_ESM.docx]
